# The Light-Driven Proton Pump Proteorhodopsin Enhances Bacterial Survival during Tough Times

**DOI:** 10.1371/journal.pbio.1000359

**Published:** 2010-04-27

**Authors:** Edward F. DeLong, Oded Béjà

**Affiliations:** 1 Department of Civil and Environmental Engineering, Massachusetts Institute of Technology, Cambridge, Massachusetts, United States of America; 2 Department of Biological Engineering, Massachusetts Institute of Technology, Cambridge, Massachusetts, United States of America; 3 Faculty of Biology, Technion - Israel Institute of Technology, Technion City, Haifa, Israel

## Abstract

Recently, it has been discovered that many microorganisms previously thought to be light-independent actually make use of sunlight for growth and survival. Newly reported work suggests some of the specific mechanisms involved.

Some microorganisms contain proteins that can interact with light and convert it into energy for growth and survival, or into sensory information that guides cells towards or away from light. The simplest energy-harvesting photoproteins are the rhodopsins, which consist of a single, membrane-embedded protein covalently bound to the chromophore retinal (a light-sensitive pigment) [1]. One class of archaeal photoproteins (called bacteriorhodopsin) was shown to function as a light-driven proton pump, generating biochemical energy from light [2],[3]. For many years, these light-driven proton pumps were thought to be found only in relatively obscure Archaea living in high salinity.

Ten years ago, a new type of microbial rhodopsin (proteorhodopsin) was discovered in marine planktonic bacterial assemblages [4]. This proteorhodopsin was co-localized on a large genome fragment containing the small subunit ribosomal RNA gene, which identified its genomic source—an uncultured gammaproteobacterium (of the SAR86 bacterial lineage). Further work showed that proteorhodopsin could be expressed in *Escherichia coli*, producing light-driven proton pumping, with photocycle kinetics similar to archaeal bacteriorhodopsins [4]. Soon, proteorhodopsins were detected in other populations of planktonic marine bacteria. These photoproteins were specifically “tuned” to absorb the wavelengths of light found in the surrounding environment—green light in surface waters and blue light at greater depths in the water column [5].

Work over the past decade shows that proteorhodopsins and related light-driven proton pumps are not an exception, but rather the rule for microbial inhabitants of sunny environments like the upper ocean (see Table 1 and Box 1). Based on genomic survey data, proteorhodopsins occur in an estimated 13% to 80% of marine bacteria and archaea in oceanic surface waters [7],[8]. Given that microbial cell densities can approach one billion microbes per litre, the potential influence of proteorhodopsin-based light-driven energy flux in ocean ecosystems is significant but still difficult to quantify directly. Proteorhodopsins add substantially to the list of phototrophs (organisms that can use light as an energy source) known to inhabitate ocean surface waters, such as oxygenic and anoxygenic chlorophyll-based phototrophs [6],[7].

**Box 1. A Decade of Proteorhodopsin Milestones**

**2000** •First proteorhodopsin gene found in uncultured SAR86 using metagenomics; proteorhodopsin light-driven proton pump activity confirmed in heterologous *E. coli* cells [4].

**2001** •Proteorhodopsin presence confirmed directly in the ocean using laser flash photolysis [5].

**2003** •Proteorhodopsin genes also found in other bacterial groups [8].

**2004** •Enormous diversity of proteorhodopsin genes found in the Sargasso Sea using metagenomics [9].

**2005** •Retinal biosynthesis pathways found in metagenomic data and confirmed using *E. coli* cells [10].

   •Proteorhodopsin genes are found in ‘*Canditatus* Pelagibacter ubique’ (SAR11), the most abundant bacterium on earth; environmental SAR11 proteorhodopsin presence confirmed using metaproteomics [11].

**2006** •Proteorhodopsin genes found in uncultured marine Archaea [12].

**2007** •First indication of proteorhodopsin light-dependent growth in cultured *Flavobacteria* [13] (see Figure 1 for colony morphologies and pigmentation).

**2008** •Proteorhodopsin genes found in non-marine environments [14],[15].

**2010** •Proteorhodopsin phototrophy directly confirmed using a genetic system in marine *Vibrio* sp. [16]

**Figure 1:**
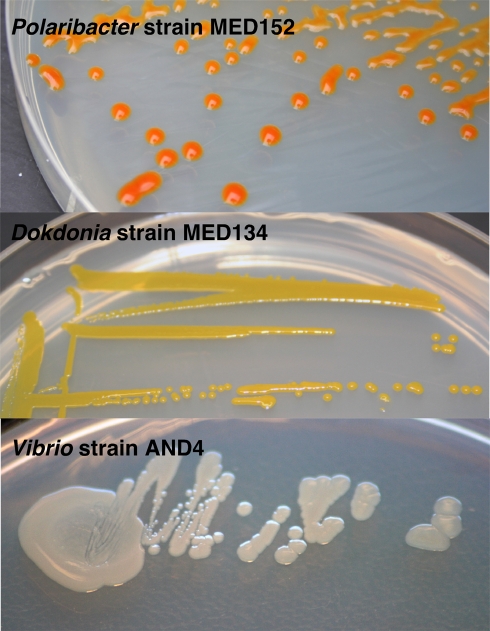
Various colony morphologies and coloration of different proteorhodopsin-containing bacteria used to study proteorhodopsin phototrophy. From top to bottom, the flavobacterium *Polaribacter dokdonensis* strain MED152 used to show proteorhodopsin light stimulated growth [13]; the flavobacterium *Dokdonia donghaensis* strain MED134 used to show proteorhodopsin light stimulated CO_2_-fixation [23]; and *Vibrio* strain AND4 used to show proteorhodopsin phototrophy [16]; note the lack of detectable pigments in *Vibrio* strain AND4. However, when these vibrio cells are pelleted, they do show a pale reddish color, which is the result of proteorhodopsin pigments presence in their membranes. Photos are courtesy of Jarone Pinhassi.

**Table 1:** Marine bacterial isolates and genome fragments containing proteorhodopsins.

Organism	Strain	General Group	Reference
**Genomes**			
Methylophilales	HTCC2181	*Betaproteobacteria*	GBMF
Rhodobacterales sp.	HTCC2255	*Alphaproteobacteria*	GBMF
*Vibrio angustum*	S14	*Gammaproteobacteria*	GBMF
*Photobacterium*	SKA34	*Gammaproteobacteria*	GBMF
*Vibrio harveyi*	ATCC BAA-1116	*Gammaproteobacteria*	GenBank # CP000789
Marine gamma	HTCC2143	*Gammaproteobacteria*	GBMF
Marine gamma	HTCC2207	*Gammaproteobacteria*	GBMF
*Cand.* P. ubique	HTCC1002	*Alphaproteobacteria*	GBMF
*Cand.* P. ubique	HTCC1062	*Alphaproteobacteria*	[26]
*Rhodospirillales*	BAL199	*Alphaproteobacteria*	GBMF
*Marinobacter*	ELB17	*Gammaproteobacteria*	GBMF
*Vibrio campbelli*	AND4	*Gammaproteobacteria*	GBMF
*Vibrio angustum*	S14	*Gammaproteobacteria*	GBMF
*Dokdonia donghaensis*	MED134	*Flavobacteria*	GBMF
*Polaribacter dokdonensis*	MED152	*Flavobacteria*	GBMF
*Psychroflexus*	ATCC700755	*Flavobacteria*	GBMF
*Polaribacter irgensii*	23-P	*Flavobacteria*	GBMF
Flavobacteria bacterium	BAL38	*Flavobacteria*	GBMF
**BACs and fosmids**			
HF10_05C07		*Proteobacteria*	[24]
HF10_45G01		*Proteobacteria*	[24]
HF130_81H07		*Gammaproteobacteria*	[24]
EB0_39F01		*Alphaproteobacteria*	[24]
EB0_39H12		*Proteobacteria*	[24]
EB80_69G07		*Alphaproteobacteria*	[24]
EB80_02D08		*Gammaproteobacteria*	[24]
EB0_35D03		*Proteobacteria*	[24]
EB0_49D07		*Proteobacteria*	[24]
EBO_50A10		*Gammaproteobacteria*	[24]
EB0_55B11f		*Alphaproteobacteria*	[24]
EBO_41B09		*Betaproteobacteria*	[24]
HF10_19P19		*Proteobacteria*	[17]
HF10_25F10		*Proteobacteria*	[17]
HF10_49E08		*Planctomycetes*	[24]
HF10_12C08		*Alphaproteobacteria*	[24]
HF10_29C11		*Euryarchaea*	[24]
MED13K09		unknown	[10]
MED18B02		unknown	[10]
MED35C06		unknown	[10]
MED42A11		unknown	[10]
MED46A06		unknown	[10]
MED49C08		unknown	[10]
MED66A03		unknown	[10]
MED82F10		unknown	[10]
MED86H08		unknown	[10]
RED17H08		unknown	[10]
RED22E04		unknown	[10]
eBACHOT4E07		*Gammaproteobacteria*	[25]
EBAC20E09		*Gammaproteobacteria*	[25]
HOT2C01		unknown	[8]
EBAC31A08		*Gammaproteobacteria*	[4]
ANT32C12		unknown	[8]
HF70_39H11_ArchHighGC		unknown	[12]
HF10_3D09_mediumGC		unknown	[12]
HF70_19B12_highGC		unknown	[12]
HF70_59C08		unknown	[12]

Marine microbial isolates and large genome fragments from the environment GBMF, microbial genomes sequenced as part of the Gordon and Betty Moore Foundation microbial genome sequencing project (http://www.moore.org/microgenome), found to encode proteorhodopsin genes. The list includes whole genome sequences from a wide array of cultivated marine microorganisms (Genomes), as well as cloned large DNA fragments (BACs and fosmids) recovered directly from the environment.

The proteorhodopsin proteins retain their native structure and function in *E. coli* membranes, an ability not shared by their close homologues, bacteriorhodopsins. Therefore, proteorhodopsins expressed in *E. coli* are very useful for probing rhodopsin structure and function [7],[18]–[21]. The relative ease of working with proteorhodopsins in *E. coli* helped to dissect their light-dependent proton pump activity using definitive biophysical assays [19],[22]–[25] and their potential role in phototrophy [17],[18] (see Figure 2 for an artist's rendition of the fundamental arrangement of proteorhodopsin in the cell membrane). However, since all this work relied on the heterologous *E. coli* system, the specific physiological roles and adaptive strategies of native marine bacteria that contain proteorhodopsin still needs to be better described.

**Figure 2:**
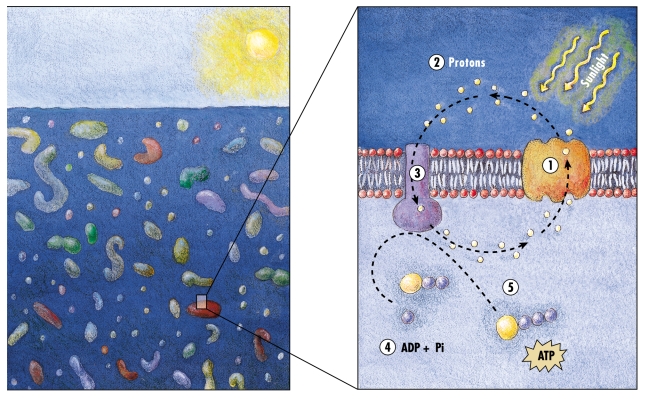
An artist's rendition of the fundamental arrangement of proteorhodopsin in the cell membrane. Left panel: a cartoon (not to scale) of planktonic bacteria in the ocean water column. Right panel: a simple view of one potential proteorhodopsin energy circuit. (1) Proteorhodopsin – uses light energy to translocate protons across the cell membrane. (2) Extracellular protons – the excess extracellular protons create a proton motive force, that can energetically drive flagellar motility, transport processes, or ATP synthesis in the cell. (3) Proton-translocating ATPase – a multi-protein membrane-bound complex that can utilize the proton motive force to synthesize 5. Adenosine triphosphate (ATP, a central high energy biochemical intermediate for the cell) from 4. Adenosine triphosphate (ADP, a lower energy biochemical intermediate). Illustration by Kirsten Carlson, © MBARI 2001.

Cultivation-independent genomic surveys (e.g., “metagenomics”) revealed proteorhodopsin presence and diversity, and heterologous expression in *E. coli* demonstrated many of its functional properties. Access to cultivable marine bacteria that contain proteorhodopsin, however, would be very useful to further characterize its native function in diverse physiological contexts.

Whole-genome sequencing then came to the rescue. The Gordon and Betty Moore Foundation (GBMF) Microbial Genome Sequencing Project (http://www.moore.org/microgenome) unexpectedly revealed that many culturable marine bacteria submitted for sequencing (including *Pelagibacter* spp., *Vibrio* spp., and *Flavobacteria* isolates) in fact possessed proteorhodopsin genes (Table 1). What can these proteorhodopsin-containing isolates tell us? Experiments with the proteorhodopsin-containing isolate ‘*Cand.* P. ubique’ (a member of the SAR11 bacterial lineage, the most abundant bacterial group in the ocean [19],[20]) showed no significant light enhancement of growth rate or yield [11]. Later, however, Gómez-Consarnau and colleagues [13] showed light-dependent growth at low-carbon concentrations in marine flavobacteria. However since these flavobacteria are difficult to manipulate genetically, a direct relationship between the proteorhodopsin protein and light-dependent growth could not be definitively shown.

Now, a new study by the same research group led by Jarone Pinhassi, published in this issue of *PLoS Biology* [16], definitively demonstrates one role for proteorhodopsin in a light-dependent adaptive strategy. In a series of experiments, they showed that marine *Vibrio* cells survived starvation much better in the light than in the dark. Since vibrios are amenable to genetic manipulation, Gómez-Consarnau et al. could construct a strain where the proteorhodopsin gene was deleted. In the absence of proteorhodopsin, light-dependent starvation survival was abolished, and then restored when proteorhodopsin was supplied in trans.

Gómez-Consarnau et al. have conclusively demonstrated for the first time at least one specific physiological role for proteorhodopsin in a native marine bacterium [16]. Considering the abundance of proteorhodopsins (estimated to be present in 80% of marine bacteria in some waters [7]) and the possibility of lateral gene transfer [12], the potential influence of even this one adaptive strategy on marine bacterioplankton could be substantial given the “feast or famine” existence experienced by many of these microbes. The *Vibrio*/proteorhodopsin model system is likely to reveal further secrets on the nature and function of proteorhodopsin photosytems in bacteria that are usually (but erroneously) considered as strict heterotrophs not capable of utilizing light at all.

While this study [16] adds an important new result, it certainly does not solve the whole puzzle of proteorhodopsin photophysiology. Considering the staggering variety of genetic, physiological and environmental contexts in which proteorhodopsin and related photoproteins are found, a great variety of light-dependent adaptive strategies are likely to occur in the natural microbial world. For example, in 2005, a new type of bacterial rhodopsin (xanthorhodopsin) was discovered in the salt-loving bacterium *Salinibacter ruber* [28]. Xanthorhodopsin is a proton-pumping retinal protein/carotenoid complex in which the carotenoid salinixanthin functions as a light-harvesting antenna, transferring energy to the rhodopsin/retinal complex [21]. It was recently suggested that the ability of rhodopsins to bind salinixanthin depends on a single glycine amino acid [30], suggesting that other recently identified retinal proteins (e.g., proteorhodopsins) might also interact with carotenoid antennas, since some possess the identical homologous glycine residue.

What questions remain to be tackled for the second decade of research on proteorhodopsin photophysiology? Maintenance of energy charge during respiratory stress or starvation, the most likely physiological mechanism explaining the results of Gómez-Consarnau et al. [16], is just one example of a life history strategy benefitting from proteorhodopsin. As Martinez and colleagues pointed out [17], in different physiological, ecological, phylogenetic, and genomic contexts, proteorhodopsin activity may benefit microbes in a variety of ways. Besides producing ATP from the light-generated proton gradient, flagellar motility and active transport of solutes into or out of the cell can make use of the proton motive force generated by proteorhodopsins [17]. Heterotrophs adapted to either high or low nutrient concentrations are known to contain and express proteorhodopsins. Whether high versus low nutrient adapted bacteria exhibit life-style–specific patterns of proteorhodopsin photophysiology remains to be determined. Already it seems clear that two different high-nutrient–utilizing bacteria containing proteorhodopsin (vibrios and flavobacteria) exhibit fundamentally different light-dependent growth strategies [13],[16]. Understanding the diversity of interactions among proteorhodopsin-containing organisms in natural communities represents yet another layer of complexity [22]. Finally, obtaining quantitative estimates of the total contribution of proteorhodopsin photosystems to the overall energy flux in microbial food webs is a worthy goal, but extremely challenging. For chlorophyll-based oxygenic photosythesizers, fluorescence-based assays, carbon dioxide uptake experiments, and oxygen evolution measurements to constrain energy garnered from sunlight are readily available. In contrast, light-dependent activity assays are not simple, nor straightforwardly interpretable for proteorhodopsin-containing microorganisms. The dizzying array of phylogenetic and physiological contexts in which proteorhodopsins are found (Table 1) also confounds any simple, universal approaches for quantifying their energetic contributions in situ. Nevertheless, the future is bright for both basic understanding as well as technological applications of proteorhodopsins and the microbes that contain them. The increasing availability of cultivable and readily manipulated model systems, along with increasingly more sophisticated in situ studies in the environment, promise to shed further light on the structure, function, and ecological significance of these ubiquitous and fascinating photoproteins.
