# Correction: The Light-Driven Proton Pump Proteorhodopsin Enhances Bacterial Survival during Tough Times

**DOI:** 10.1371/annotation/9505d865-7e1e-4f1e-84a0-f568d8552414

**Published:** 2012-05-03

**Authors:** Edward F. DeLong, Oded Béjà

In this paragraph:

[16] should be [21]

[12] should be [22]

