# Correction: The Light-Driven Proton Pump Proteorhodopsin Enhances Bacterial Survival during Tough Times

**DOI:** 10.1371/annotation/a260c562-9d55-452e-84fa-f33ce59b9704

**Published:** 2012-05-03

**Authors:** Edward F. DeLong, Oded Béjà

In this box:

[8] should be [31]

[9] should be [32]

[10] should be [6]

[11] should be [19]

[12] should be [22]

[13] should be [20]

[14,15] should be [33,34]

[16] should be [21]

