# Correction: The Light-Driven Proton Pump Proteorhodopsin Enhances Bacterial Survival during Tough Times

**DOI:** 10.1371/annotation/a9234cc3-a14f-4c3f-8be6-645bfeca2b6c

**Published:** 2012-05-03

**Authors:** Edward F. DeLong, Oded Béjà

In this paragraph:

[7,18-21] should be [6,9-12]

[19,22-25] should be [10,13-16]

[17,18] should be [11,12]

