# Correction: The Light-Driven Proton Pump Proteorhodopsin Enhances Bacterial Survival during Tough Times

**DOI:** 10.1371/annotation/6830e1ff-b565-4054-bae6-02939f625cfe

**Published:** 2012-05-03

**Authors:** Edward F. DeLong, Oded Béjà

In this paragraph:

[19,20] should be [17,18]

[11] should be [19]

[13] should be [20]

