# Correction: The Light-Driven Proton Pump Proteorhodopsin Enhances Bacterial Survival during Tough Times

**DOI:** 10.1371/annotation/fe6ae2ff-6778-4581-8d52-4aba3534c99f

**Published:** 2012-05-03

**Authors:** Edward F. DeLong, Oded Béjà

In this table:

[26] should be [28]

[24] should be [29]

[17] should be [11]

[24] should be [29]

[10] should be [6]

[25] should be [30]

[8] should be [31]

[12] should be [22]

