# Correction: The Light-Driven Proton Pump Proteorhodopsin Enhances Bacterial Survival during Tough Times

**DOI:** 10.1371/annotation/571dcbb4-cac9-4c84-9f9e-27cc87ceed0e

**Published:** 2012-05-03

**Authors:** Edward F. DeLong, Oded Béjà

In this paragraph:

[7,8] should be [6,7]

[6,7] should be [7,8]

