# Correction: The Light-Driven Proton Pump Proteorhodopsin Enhances Bacterial Survival during Tough Times

**DOI:** 10.1371/annotation/05d3c1ef-0aad-4592-ab8d-6863fbdf73f9

**Published:** 2012-05-03

**Authors:** Edward F. DeLong, Oded Béjà

In this paragraph:

[16] should be [21]

[28] should be [23]

[21] should be [24]

[30] should be [25]

