# Correction: The Light-Driven Proton Pump Proteorhodopsin Enhances Bacterial Survival during Tough Times

**DOI:** 10.1371/annotation/48a18f09-0a1a-469e-8c0d-b352e80200bc

**Published:** 2012-05-03

**Authors:** Edward F. DeLong, Oded Béjà

In this paragraph:

[16] should be [21]

[17] should be [11]

[17] should be [11]

[13,16] should be [20,21]

[22] should be [26]

