# Correction: The Light-Driven Proton Pump Proteorhodopsin Enhances Bacterial Survival during Tough Times

**DOI:** 10.1371/annotation/65474650-89c5-4b56-80e8-6e513347d3d7

**Published:** 2012-05-03

**Authors:** Edward F. DeLong, Oded Béjà

In this paragraph:

[13] should be [20]

[23] should be [27]

[16] should be [21]

