# Correction: The Light-Driven Proton Pump Proteorhodopsin Enhances Bacterial Survival during Tough Times

**DOI:** 10.1371/annotation/52943e21-9d9d-4a2a-ba23-942bd6f01716

**Published:** 2012-05-03

**Authors:** Edward F. DeLong, Oded Béjà

A number of references and their citations were numbered incorrectly. The corrected reference list is below, and each paragraph is marked with the correct reference citations. The authors wish to apologize for any inconvenience to readers.

1. Spudich JL, Jung KH (2005) Microbial Rhodopsins: Phylogenetic and Functional Diversity. In: Briggs WR, Spudich JL, editors. Handbook of Photosensory Receptors. Weinheim: WILEY-VCH Verlag GmbH & Co. KGaA. pp. 1-24.

2. Oesterhelt D, Stoeckenius W (1971) Rhodopsin-like protein from the purple membrane of Halobacterium halobium. Nat New Biol 233: 149-152.

3. Spudich JL, Yang CS, Jung KH, Spudich EN (2000) Retinylidene proteins: Structures and Functions from Archaea to Humans. Annu Rev Cell Dev Biol 16: 365-392.

4. Béjà O, Aravind L, Koonin EV, Suzuki MT, Hadd A, et al. (2000) Bacterial rhodopsin: evidence for a new type of phototrophy in the sea. Science 289: 1902-1906.

5. Béjà O, Spudich EN, Spudich JL, Leclerc M, DeLong EF (2001) Proteorhodopsin phototrophy in the ocean. Nature 411: 786-789.

6. Sabehi G, Loy A, Jung KH, Partha R, Spudich JL, et al. (2005) New insights into metabolic properties of marine bacteria encoding proteorhodopsins. PLoS Biol 3: e173.

7. Moran MA, Miller WL (2007) Resourceful heterotrophs make the most of light in the coastal ocean. Nat Rev Microbiol 5: 792-800.

8. Karl DM (2002) Hidden in a sea of microbes. Nature 415: 590-591.

9. Man D, Wang W, Sabehi G, Aravind L, Post AF, et al. (2003) Diversification and spectral tuning in marine proteorhodopsins. EMBO J 22: 1725-1731.

10. Man-Aharonovich D, Sabehi G, Sineshchekov OA, Spudich EN, Spudich JL, et al. (2004) Characterization of RS29, a blue-green proteorhodopsin variant from the Red Sea. Photochem Photobiol Sci 3: 459-462.

11. Martinez A, Bradley AS, Waldbauer J, Summons RE, DeLong EF (2007) Proteorhodopsin photosystem gene expression enables photophosphorylation in heterologous host. Proc Natl Acad Sci U S A 104: 5590-5595.

12. Walter JM, Greenfield D, Bustamante C, Liphardt J (2007) Light-powering Escherichia coli with proteorhodopsin Proc Natl Acad Sci U S A 104: 2408-2412 

13. Wang WW, Sineshchekov OA, Spudich EN, Spudich JL (2003) Spectroscopic and photochemical characterization of a deep ocean proteorhodopsin. J Biol Chem 278: 33985-33991.

14. Sineshchekov OA, Spudich JL (2004) Light-induced intramolecular charge movements in microbial rhodopsins in intact E. coli. Photochem Photobiol Sci 3: 548-554.

15. Bergo VB, Sineshchekov OA, Kralj JM, Partha R, Spudich EN, et al. (2009) His-75 in proteorhodopsin, a novel component in light-driven proton translocation by primary pumps. J Biol Chem 284: 2836-2843.

16. Friedrich T, Geibel S, Kalmbach R, Chizhov I, Ataka K, et al. (2002) Proteorhodopsin is a Light-driven Proton Pump with Variable Vectoriality. J Mol Biol 321: 821-838.

17. Rappé MS, Connon SA, Vergin KL, Giovannoni SJ (2002) Cultivation of the ubiquitous SAR11 marine bacterioplankton clade. Nature 418: 630-633.

18. Morris RM, Rappé MS, Connon SA, Vergin KL, Siebold WA, et al. (2002) SAR11 clade dominates ocean surface bacterioplankton communities. Nature 420: 806-810.

19. Giovannoni SJ, Bibbs L, Cho J-C, Stapels MD, Desiderio R, et al. (2005) Proteorhodopsin in the ubiquitous marine bacterium SAR11. Nature 438: 82-85.

20. Gómez-Consarnau L, González JM, Coll-Lladó M, Gourdon P, Pascher T, et al. (2007) Light stimulates growth of proteorhodopsin-containing marine Flavobacteria. Nature 445: 210-213.

21. Gómez-Consarnau L, Akram N, Lindell K, Pedersen A, Neutze R, et al. (2010) Proteorhodopsin phototrophy confers enhanced survival of marine bacteria during starvation. PLoS Biol 8: e1000358.

22. Frigaard N-U, Martinez A, Mincer TJ, DeLong EF (2006) Proteorhodopsin lateral gene transfer between marine planktonic Bacteria and Archaea. Nature 439: 847-850.

23. Balashov SP, Imasheva ES, Boichenko VA, Anton J, Wang JM, et al. (2005) Xanthorhodopsin: a proton pump with a light-harvesting carotenoid antenna. Science 309: 2061-2064.

24. Lanyi JK, Balashov SP (2008) Xanthorhodopsin: a bacteriorhodopsin-like proton pump with a carotenoid antenna. Biochim Biophys Acta 1777: 684-688.

25. Imasheva ES, Balashov SP, Choi AR, Jung KH, Lanyi JK (2009) Reconstitution of Gloeobacter violaceus rhodopsin with a light-harvesting carotenoid antenna. Biochemistry 48: 10948-10955.

26. Lami R, Cottrell MT, Campbell BJ, Kirchman DL (2009) Light-dependent growth and proteorhodopsin expression by Flavobacteria and SAR11 in experiments with Delaware coastal waters. Environ Microbiol 11: 3201-3209.

27. González JM, Fernandez-Gomez B, Fernandez-Guerra A, Gomez-Consarnau L, Sanchez O, et al. (2008) Genome analysis of the proteorhodopsin-containing marine bacterium Polaribacter sp. MED152 (Flavobacteria). Proc Natl Acad Sci U S A 105: 8724-8729.

28. Giovannoni SJ, Tripp HJ, Givan S, Podar M, Vergin KL, et al. (2005) Genome streamlining in a cosmopolitan oceanic bacterium. Science 309: 1242-1245.

29. McCarren J, DeLong EF (2007) Proteorhodopsin photosystem gene clusters exhibit co-evolutionary trends and shared ancestry among diverse marine microbial phyla. Environ Microbiol 9: 846-858.

30. Sabehi G, Béjà O, Suzuki MT, Preston CM, DeLong EF (2004) Different SAR86 subgroups harbour divergent proteorhodopsins. Environ Microbiol 6: 903-910.

31. de la Torre JR, Christianson L, Béjà O, Suzuki MT, Karl D, et al. (2003) Proteorhodopsin genes are widely distributed among divergent bacterial taxa. Proc Natl Acad Sci U S A 100: 12830-12835.

32. Venter JC, Remington K, Heidelberg J, Halpern AL, Rusch D, et al. (2004) Environmental genome shotgun sequencing of the Sargasso Sea. Science 304: 66-74.

33. Atamna-Ismaeel N, Sabehi G, Sharon I, Witzel K-P, Labrenz M, et al. (2008) Widespread distribution of proteorhodopsins in freshwater and brackish ecosystems. ISME J 2: 656-662.

34. Sharma AK, Zhaxybayeva O, Papke RT, Doolittle WF (2008) Actinorhodopsins: proteorhodopsin-like gene sequences found predominantly in non-marine environments. Environ Microbiol 10: 1039-1056.

